# Preclinical Research on Cinnamic Acid Derivatives for the Prevention of Liver Damage: Promising Therapies for Liver Diseases

**DOI:** 10.3390/biomedicines13051094

**Published:** 2025-04-30

**Authors:** Liseth Rubí Aldaba-Muruato, Brayan Escalante-Hipólito, Aldo Yoshio Alarcón-López, Pablo A. Martínez-Soriano, Enrique Angeles, José Roberto Macías-Pérez

**Affiliations:** 1 Laboratorio de Ciencias Biomédicas, Facultad de Estudios Profesionales Zona Huasteca, Universidad Autónoma de San Luis Potosí, Ciudad Valles 79060, Mexico; liseth.aldaba@uaslp.mx (L.R.A.-M.); a276864@alumnos.uaslp.mx (B.E.-H.); 2 Laboratorio de Química Teórica y Medicinal, Departamento de Ciencias Químicas, Facultad de Estudios Superiores Cuautitlán, Universidad Nacional Autónoma de México, Cuautitlán Izcalli 54750, Mexico; aldo.yoshio@cuautitlan.unam.mx (A.Y.A.-L.); parturomart@cuautitlan.unam.mx (P.A.M.-S.); angeles@unam.mx (E.A.)

**Keywords:** liver damage, cinnamic acid derivatives, LQM717, LQM755, CCl_4_

## Abstract

**Background:** Liver diseases are a global health issue with an annual mortality of 80,000 patients, mainly due to complications that arise during disease progression, as effective treatments are lacking. **Objectives:** This study evaluated the hepatoprotective effects of two derivatives of cinnamic acid, LQM717 and LQM755, in a murine model of acute liver damage induced by carbon tetrachloride (CCl_4,_ 4 g/kg, single dose p.o.). **Methods:** Male Wistar rats were pretreated with five doses of LQM717 (20 mg/kg i.p.) or LQM755 (equimolar dose), starting 2 days before inducing hepatotoxic damage with CCl_4_. **Results:** The key parameters of hepatocellular function and damage showed significant increases in ALT, ALP, GGT, and total and direct bilirubin in rats intoxicated with CCl_4_, with decreased liver glycogen and serum albumin. Macroscopic and microscopic liver examinations revealed reduced inflammation, necrosis, and steatosis in animals pretreated with LQM717 or LQM755. Hepatomegaly was observed only in the LQM717 + CCl_4_ group. LQM755 statistically provided partial protection against increases in ALT and ALP and completely prevented elevations in GGT and total and direct bilirubin. LQM755 completely prevented albumin reduction, while LQM717 only partially prevented it. Both compounds partially prevented glycogen depletion. Bioinformatic analysis identified 32 potential liver protein targets for LQM717 and 36 for LQM755. **Conclusions:** These findings suggest that LQM717 and LQM755 have significant hepatoprotective effects against CCl_4_-induced acute liver injury, providing information for future studies in other acute and chronic models, as well as to elucidate their mechanisms of action.

## 1. Introduction

Liver diseases are responsible for more than two million deaths annually, accounting for 4% of all deaths worldwide [1,2]. Approximately 80 million individuals worldwide are estimated to be afflicted with various forms of liver disease. The economic burden of this condition is substantial, with costs amounting to EUR 27 billion in the 12 member states of the European Union in 2008, USD 19.2 billion in the United States in 2004, AUD 2.5 billion in Australia in 2010, and CNY 35 billion in China in 2006. The elevated risk of liver failure, which can be fatal, further exacerbates this situation. Furthermore, in recent years, the incidence of liver disease has increased both in the Western world and in several developing areas [3,4,5,6].

The most common liver diseases include viral hepatitis, cirrhosis, hepatocellular carcinoma (HCC), nonalcoholic fatty liver disease (NAFLD), fatty liver, hepatitis, hepatocirrhosis, hepatoma, biliary cysts, cholecystitis, and biliary cholangitis. The increasing incidence of these diseases is partly because, despite advances in the search for new treatments, effective drugs have not yet been developed. There are some treatments of HCC, such as Sorafenib, Lenvatinib, Regorafenib, Cabozantinib, and Ramucirumab, which have been shown to have antineoplastic activities. Nevertheless, liver transplantation (LT) remains the treatment of choice for patients with advanced liver damage, although it is associated with posttransplant complications and long waiting times [7,8,9,10,11,12,13,14].

Over the years, an expanding body of research has delved into the therapeutic properties of cinnamic acid derivatives (CADs). These compounds have demonstrated a broad spectrum of activities, including anti-inflammatory, antioxidant, antiviral, antiplatelet, antifungal, antibacterial, and anticancer properties. Cinnamic acid and its hydroxylated and methoxylated analogs have been found in numerous natural environments, either in their free or conjugated state, through ester links, the C1 ether of the aromatic ring, or O-glycoside links [15,16,17].

Cinnamic acid and the phenethyl ester of caffeic acid (CAPE) are intermediates and end products in many secondary metabolic pathways in plants. Many of the hydroxystyrene derivatives present in plants are generated by the hydroxylation of cinnamic acid, formic acid, and alanine. In combination with other constituent units, such as glucose, CADs are found in plants and form basic lignocellulosic structures. The *p*-coumarate units in CADs are used specifically in plants for the construction of lignin polymers, which are present in hardwoods, grasses, and other plants, such as mosses, in various chemical forms. Numerous CADs have been shown to have chemotherapeutic and chemoprophylactic value, such as LQM755, which has a cytotoxic effect on a human gastric adenocarcinoma cell-line [18], and LQM717, which reduces the formation of liver cancer in the resistant hepatocyte modified model in rats [19]. These products, which are consumed through plant-based diets, play important physiological roles in the human body. Furthermore, phenolic compounds derived from hydroxycinnamic acid, which are present in high concentrations in tea, play a key role in the potential health benefits of these compounds. Some specialty teas have been shown to improve liver function. Similarly, components such as cinnamic acids and hydroxystyrenes have been identified in products derived from bees and vegetable oils [20,21,22].

Several in vitro investigations have demonstrated that CADs possess the capacity to mitigate the impacts of various hepatotoxic agents [23] in experimental models, predominantly concentrated on hepatocellular carcinomas, or liver damage induced by substances such as paracetamol [24] or carbon tetrachloride (CCl_4_) [25]. However, studies have focused on other virus-driven cell signaling mechanisms that contribute to liver disease. For example, Li and colleagues reported that ursodeoxycholic acid–cinnamic acid hybrids inhibit inflammation Akt/NF-κB and MAPK signaling pathways [26].

**Figure 1:**
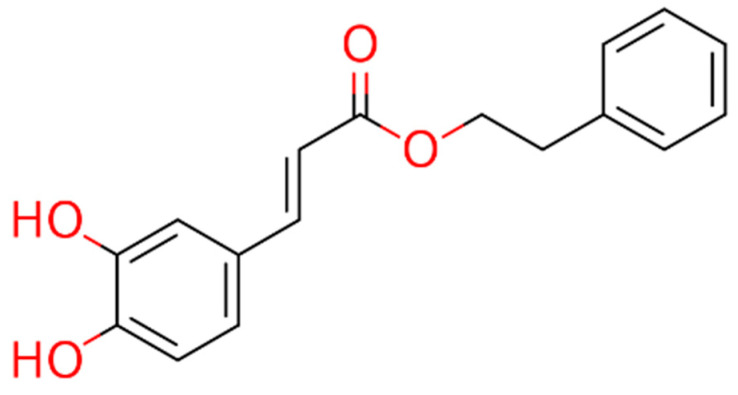
Chemical structure of CAPE.

These observations prompted the idea of using cinnamic acid to generate derivatives, specifically amides, that could offer metabolic advantages over esters, such as CAPE (Figure 1), as cinnamic acid is not an esterase substrate and therefore has greater stability in biological systems.

Our research group has synthesized several derivatives of cinnamic acid, reporting significant effects on gastric cancer [18,19,27] and viral infections [unpublished results]. In this work, we present the hepatoprotective activity of two derivatives, LQM717 and LQM755, which have shown promising results (Table 1).

## 2. Materials and Methods

### 2.1. Animal Testing

Male Wistar rats (Rattus norvegicus, *n* = 31) weighing between 280 and 300 g were used. All experimental procedures were approved by the Research Ethics Committee of Facultad de Estudios Profesionales de Zona Huasteca, Ciudad Valles, México, and were carried out in accordance with international guidelines and technical specifications for the production, care, and use of animals established in the official Mexican standard NOM-062-ZOO-1999 [28]. The rats were maintained under controlled conditions, with a standard diet (LabDiet 5008^®^, PMI, St. Louis, MO, USA), free access to drinking water, a temperature of 22 ± 2 °C, a relative humidity of 50–60%, and a 12 h light–dark cycle.

### 2.2. Chemical Products

#### Chemicals

The reagents used in this investigation included carbon tetrachloride (CCl4, Malincrodt, St. Louis, MO, USA), mineral oil (Azumex, Puebla, Mexico), acetone and formaldehyde (Hycel, Jalisco, Mexico), xylene and sodium hydroxide (JT Baker, Madrid, Spain), ammonia water (CF-Industries, Deerfield, IL, USA), glycine (Santa Cruz Biotechnology, Dallas, TX, USA), potassium dichromate (Karal, Guanajuato, Mexico), basic fuchsin (Golden Bell, Jalisco, Mexico), and sodium metabisulfite (Karal, Guanajuato, Mexico). From Jalmek (NL, Mexico), hydrochloric acid, potassium hydroxide, sulfuric acid, magnesium chloride, Harris hematoxylin, yellowish eosin, and picric acid were used. Merck chemicals (Darmstadt, Germany) include *p*-nitrophenylphosphate, 2,4-dinitrophenylhydrazine, DL-alanine, p-nitrophenol, α-ketoglutaric acid, sodium pyruvate, p-nitroaniline, gamma-glutamyl-p-nitroanilide, dextrose, anthrone, phosphate disodium, monosodium phosphate, ketamine, xylazine, silane (silicon hydride), dimethylsulfoxide (DMSO, ACS reagent ≥ 99.9%), and Entellan.

### 2.3. Preparation of LQM717 and LQM755

#### 2.3.1. Experimental Section

The cinnamic acid analog 4-phenoxycinnamic acid, which is not commercially available, was synthesized via Knövenagel–Döbner condensation using piperidine as a catalyst and glacial acetic acid as a solvent. Phenylacetic acid was the only reagent purchased from Sigma-Aldrich Co, SLM, St. Louis, MO, USA.

#### 2.3.2. Procedure for the Synthesis of 3-(4-Phenoxyphenyl)-2-Propenoic Acid

**Figure 2:**
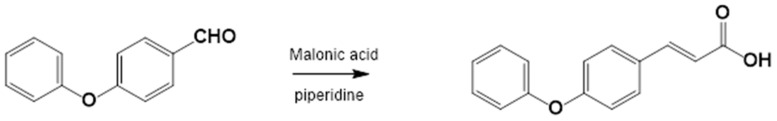
3-(4-Phenoxyphenyl)-2-propenoic acid. Yield: 85%; mp 156–157 °C. IR (diamond, cm^−1^): νmax 1588, 3031, 3200–2200. ^1^H NMR (300 MHz, CDCl_3_): δ 6.30 (1H, d), 6.90 (5H, m), 7.24 (4H, m), 7.64 (1H, d, J = 15.6 Hz), 8.95 (1H, s). ^13^C NMR (75 MHz, CDCl_3_): δ 116.02, 117.02, 117.32, 121.61, 125.69, 128.21, 128.91, 144.64, 155.32, 156.13, 169.64.

Equimolar amounts of 4-Phenoxybenzaldehyde and malonic acid were placed in a round-bottom flask along with piperidine (1.25 equiv.) and glacial acetic acid (1.77 equiv.) (Figure 2). The reaction mixture was heated under reflux at 140–160 °C and monitored by TLC (hexane:EtOAc, 80:20). Upon completion, the reaction mixture was poured into ice-cold water to precipitate the product, which was then filtered and washed with water (3 × 100 mL). The crude product was recrystallized from EtOAc. If precipitation did not occur, the reaction mixture was extracted with EtOAc (5 × 50 mL), washed with brine (3 × 30 mL), dried over anhydrous sodium sulfate, and recrystallized.

#### 2.3.3. Amide Synthesis

**Figure 3:**
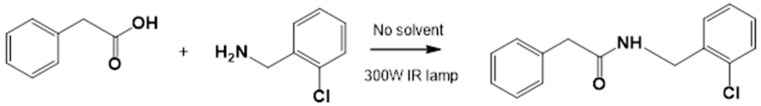
LQM717—N-(2-Chlorobenzyl)-2-phenylacetamide (11). Yield: 3.9 g (79.83%); mp 118–120 °C. IR (diamond, cm^−1^): νmax 1546, 2923, 3274. ^1^H NMR (300 MHz, CDCl_3_, Me_4_Si): δ 3.63 (2H, s), 4.46 (2H, d, J = 4.0 Hz), 5.90 (1H, br s, NH), 7.29 (9H, m). ^13^C NMR (75 MHz, CDCl_3_, Me_4_Si): δ 42.05, 44.18, 127.44, 127.85, 129.28, 129.48, 129.88, 129.89, 130.32, 133.91, 135.05, 135.86, 171.29. Elemental analysis calcd. for C_15_H_14_ClNO: C, 69.33; H, 5.43; Cl, 13.64; N, 5.39; O, 6.16. Found: C, 69.44; H, 5.05; Cl, 13.50; N, 5.71; O, 6.22.

**Figure 4:**
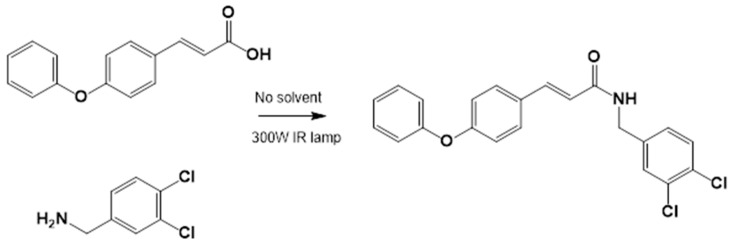
LQM755.—3-(4-Phenoxy)phenyl-N-[(3,4-dichlorophenyl)methyl]prop-2-enamide. Yield: 41.98%; mp 136 °C; white solid. IR (diamond, cm^−1^): νmax 3400–3100 (N–H), 3043.48 (C–H sp^2^), 2921.22 (C–H sp^3^), 2478.05, 2401.64, 2328.29, 1606.98 (C=O), 1495 (C=C). ^1^H NMR (300 MHz, CDCl_3_, Me_4_Si): δ 8.67 (1H, t, NH), 7.57–7.56 (1H, m), 7.55–7.54 (2H, m), 7.50 (2H, d), 7.42 (1H, d, =CH), 7.40–7.37 (1H, m), 7.26–7.24 (1H, m), 7.16–7.13 (1H, m), 7.04–7.02 (2H, m), 6.96 (2H, d), 6.55 (1H, d, =CH), 4.36 (2H, d, CH_2_). ^13^C NMR (75 MHz, CDCl_3_, Me_4_Si): δ 165.79, 158.60, 156.38, 141.37, 139.13, 131.42, 131.04, 130.71, 129.79, 128.22, 130.36, 130.03, 124.60, 129.86, 121.10, 118.92, 119.82, 41.79. Elemental analysis calcd. for C_22_H_17_Cl_2_NO_2_: C, 66.30; H, 4.30; Cl, 17.80; N, 3.52; O, 8.04. Found: C, 69.35; H, 5.05; Cl, 17.60; N, 3.49; O, 8.01.

The corresponding cinnamic acid and amine (1:1 molar ratio) were combined in a round-bottom flask and heated at 140–160 °C using a 300 W infrared lamp (Figure 3 and Figure 4). Reaction progress was monitored by TLC (hexane:EtOAc, 50:50). Upon completion, EtOAc was added to dissolve the product. Activated charcoal was added, and the mixture was filtered through diatomaceous earth (Hyflo Super Cel^®^, Merck KGaA, Darmstadt, Germany). The solvent was removed under reduced pressure, and the residue was recrystallized from EtOAc. Further purification was performed using EtOAc:hexane (90:10).

### 2.4. Preparation of LQM717 and LQM755 for Intraperitoneal Administration

The dose of LQM717 was 20 mg/kg, i.p., as in previous works [19], while the dose of LQM755 was equimolar to that of LQM717. In both cases, DMSO was used as a vehicle (Table 2). After their preparation, the compounds were immediately administered to each rat.

### 2.5. In Vivo Experimental Protocol

**Figure 5:**
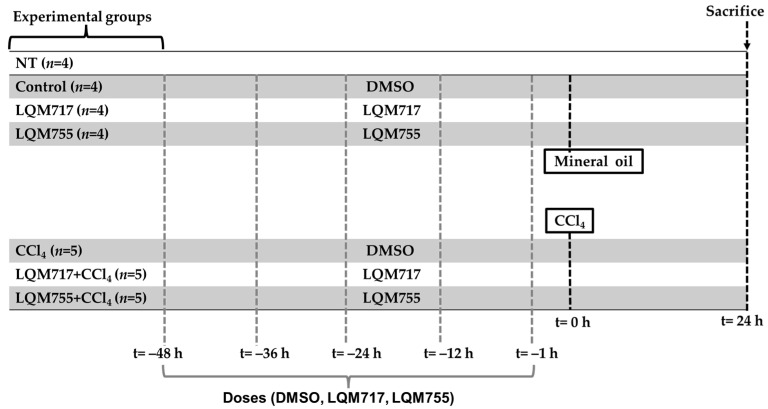
Experimental groups and dosing schedule.

The experimental rats were randomly divided into seven groups (Figure 5):

Healthy control groups:

i. NT (untreated): Without any administration or treatment.
ii. Control: Five administrations of DMSO (100 µL, i.p.) and one administration of mineral oil (250 µL/100 g, p.o.), which are the vehicles for the compounds and CCl_4_, respectively.
iii. LQM717: Rats pretreated with five doses of LQM717 and one administration of mineral oil.
iv. LQM755: Rats pretreated with five doses of LQM755 and one administration of mineral oil.

Groups with liver damage:

v. CCl_4_: Rats were treated five times with DMSO and were intoxicated with a sublethal dose of CCl_4_ (4 g/kg, p.o.).
vi. LQM717 + CCl_4_: Rats pretreated with five doses of LQM717 and intoxicated with a sublethal dose of CCl_4_.
vii. LQM755 + CCl_4_: Rats pretreated with five doses of LQM755 and intoxicated with a sublethal dose of CCl_4_.

The animals were sacrificed 24 h after CCl_4_ or mineral oil administration. All the animals were weighed at the beginning of the treatments and on the day of sacrifice.

The NT (i) group was not subjected to any experimental manipulation or intervention. The Control (ii) and CCl_4_ (v) groups received DMSO every 12 h for 2 days (5 times) prior to the administration of mineral oil or CCl_4_, respectively. The LQM717 (iii) and LQM717 + CCl_4_ (vi) groups were pretreated with LQM717 (20 mg/kg, i.p., every 12 h for 2 days, for a total of 5 doses) before the administration of mineral oil or CCl_4_, respectively. The LQM755 (iv) and LQM755 + CCl_4_ (vii) groups were pretreated with LQM755 (equimolar dose to LQM717, i.p., every 12 h for 2 days, for a total of 5 doses) before the administration of mineral oil or CCl_4_, respectively.

### 2.6. Sacrifice of the Animals

Experimental animals were sedated with an intraperitoneal mixture of ketamine (75 mg/kg) and xylazine (10 mg/kg) and subsequently sacrificed by exsanguination via cardiac puncture. Animal death was confirmed by the absence of a heartbeat and the cessation of thermoregulation [29]. Blood samples were centrifuged at 1200× *g* for 10 min in a Sorvell^®^ centrifuge (Waltham, MA, USA) to perform liver function tests on the serum.

### 2.7. In Situ Liver Evaluation

Macroscopic evaluation of the livers was carried out by taking photographs in situ before being extracted from the abdominal cavity. The color and texture of the livers from each experimental group were subsequently recorded.

### 2.8. Histochemical Procedures

#### 2.8.1. Paraffin Embedding Tissue Samples and Sectioning

The liver was carefully removed, immediately washed with cold sterile physiological saline, weighed, and sectioned into small pieces with a scalpel. A portion of these fragments was fixed in glass bottles with a 4% solution of p-formaldehyde in phosphate buffer at pH = 7.

Liver samples fixed with p-formaldehyde were subjected to dehydration by immersion in ethanol (70%, 85%, 96%, and ≥99%, 1 h each at 60 °C) and clearance by successive immersions in absolute ethanol:xylene (1:1) and xylene for 1 h each at 60 °C. The tissues were then impregnated with liquid paraffin at 60 °C, forming a paraffin block with the tissue inside. Subsequently, 4 µm thick sections were obtained from each tissue via a microtome (ECOSHEL 202A, Pharr, TX, USA); several sections were fixed on slides previously treated with silane.

#### 2.8.2. H&E Staining

Liver sections (4 µm) were deparaffinized at 60 °C overnight and then successively dipped in xylene, absolute ethanol:xylene (1:1), ethanol (≥99%, 96%, 80%), distilled water, hematoxylin, distilled water, acid alcohol, distilled water, ammonia water, eosin, ethanol 80%, 96%, ≥99%, absolute ethanol:xylene (1:1), and xylene. Finally, they were mounted with Entellan [30].

#### 2.8.3. PAS Staining

Liver sections (4 µm) were fixed on slides previously treated with silane, deparaffinized at 60 °C overnight, and then dipped in xylene, absolute ethanol:xylene (1:1), ethanol (96%, 80%), distilled water, periodic acid, distilled water, Schiff’s reagent (37 °C), distilled water, hematoxylin, distilled water, ethanol (80%, 96%, and ≥99%), absolute ethanol:xylene (1:1), or xylene [30,31].

#### 2.8.4. Biochemical Marker of Liver Necrosis

Liver damage was assessed by quantifying serum alanine aminotransferase (ALT) activity according to the specifications described by Reitman [32].

#### 2.8.5. Biochemical Markers of Cholestasis

Biochemical markers of cholestasis were evaluated by measuring the serum activity of alkaline phosphatase (ALP) following the protocol described by Berger and Rudolph [33]. Serum gamma-glutamyl transpeptidase (GGT) activity was determined according to the instructions of the manufacturer of the commercial SPINREACT kit (γ-GT; 1001185). Additionally, direct and total bilirubin serum concentrations were measured via the SPINREACT protocol (Bilirubin T & D-SPINREACT-1001044).

#### 2.8.6. Biochemical Markers of Liver Function

Liver function was assessed by measuring the serum albumin concentration according to the procedure indicated by the kit supplier (Albumin-SPINREACT-1001020). Furthermore, liver glycogen was assessed via the anthrone method described by Seifter and Dayton [34].

#### 2.8.7. Statistical Analysis

The results of body/liver weights and biochemical tests were subjected to statistical analysis to assess their level of significance, which was expressed as the means ± SD, through a one-way analysis of variance (ANOVA), followed by a Tukey–Kramer test with a significance of * *p* < 0.05 via GraphPad Prism 8.00 software.

#### 2.8.8. Bioinformatic Analysis

In this study, open-access bioinformatic platforms were used to predict possible protein targets in the liver that could interact with compounds LQM717 and LQM755. To achieve this, the ZINC20 page https://zinc.docking.org/ (accessed on 11 January 2024) was consulted to obtain simplified molecular input line entry system (SMILES) codes, which describe the connectivity of atoms in a molecular structure and are frequently used in bioinformatic studies [35]. With the SMILES codes of the compounds LQM717 and LQM755, possible protein targets were predicted via eight specialized online open-access platforms: SwissTarget Prediction [36] http://www.swisstargetprediction.ch (accessed on 11 January 2024), Super-PRED https://prediction.charite.de/ (accessed on 11 January 2024) [37], ChEMBL [38] https://www.ebi.ac.uk/chembl (accessed on 11 January 2024), PharmMapper [39] http://www.lilab-ecust.cn/pharmmapper/ (accessed on 11 January 2024), Pharos [40] https://pharos.nih.gov/ (accessed on 16 January 2024) the similarity ensemble approach [41] https://sea.bkslab.org/ (accessed on 16 January 2024), TargetNet [42] https://targetnet.scbdd.com (accessed on 15 January 2024), and BindingDB [43] https://www.bindingdb.org/rwd/bind/index.jsp (accessed on 16 January 2024). Possible targets whose probability value was equal to or greater than 50% (equivalent to a declared value of 0.5 on some platforms) were selected. Only those that were expressed in liver cells were subsequently selected via the Human Protein Atlas https://www.proteinatlas.org/ (accessed on 17 January 2024).

## 3. Results

### 3.1. Chemistry

#### 3.1.1. Synthesis of 4-Phenoxy Cinnamic Acid

**Figure 6:**
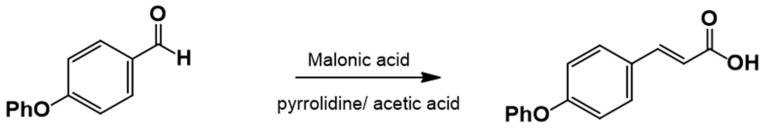
Synthesis of 4-phenoxy cinnamic acid.

The corresponding substituted 4-phenoxybenzaldehyde and malonic acid, weighed in a 1:1 ratio, were placed in a flask with piperidine (1 mL for each part of the substituted benzaldehyde, ratio of 1.25:1) and glacial acetic acid (2.5 mL for each part of the substituted benzaldehyde, ratio of 1.77:1). This flask was connected to the reflux condenser at 140–160 °C, which is the temperature necessary to perform the reaction, followed by TLC (hexane:EtOAc, 80:20). Upon completion of the reaction, ice or cold water was added to the flask until the acid precipitated; the mixture was then filtered and washed repeatedly with water (3 × 100 mL). Prior to recrystallization, the acids were dissolved in AcOEt, and anhydrous sodium sulfate was added to eliminate residual water. Recrystallization from EtOAc produces the corresponding 4-phenoxycinnamic acid (Figure 6).

#### 3.1.2. General Procedure for the Preparation of Amides

**Figure 7:**
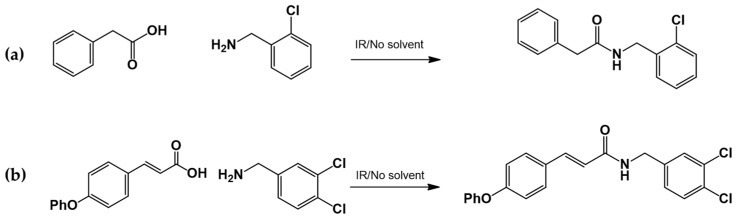
Preparation of LQM717 and LQM755. (**a**) LQM717: N-(2-Chlorobenzyl)-2-phenylacetamide, yield (3.9 g, 79.83%); mp 118 –120 °C. IR (Diamond, cm^−1^): ν max 1546, 2923, 3274. ^1^H NMR (300 MHz; CDCl_3_; Me_4_Si) δ (ppm)3.63 (2H, s), 4.46 (2H, d, J = 4.0), 5.90 (1H, br, s, NH), 7.29 (9H, m); ^13^C NMR (75 MHz; CDCl_3_; Me_4_Si) δ (ppm) 42.05 (CH_2_), 44.18 (CH_2_), 127.44, 127.85, 129.28, 129.48, 129.88, 129.89, 130.32, 133.91, 135.05, 135.86 (Ph), 171.29 (-CO-). Analysis Calc. For C_15_H_14_ClNO: C, 69.3; H, 5.4; N, 5.3. Found C, 69.35; H, 5.05; N, 5.71. (**b**) LQM755: Yield (42%), mp 135–136 °C. IR (Diamond, cm^−1^): ν max 1495, 1606, 2478, 2401, 2328, 2921, 3043, 3100–3400. ^1^H NMR (500 MHz; DMSO-D_6_; Me_4_Si) δ (ppm) 4.35 (2H, d), 6.55 (1H, d), 6.96 (2H, d), 7.03 (2H, m), 7.14 (1H, m), 7.24 (1H, m), 7.38 (1H, m), 7.42 (1H, d), 7.50 (2H, d), 7.54 (2H, m), 7.56 (1H, m), 8.66 (1H, t). ^13^C NMR (125 MHz DMSO-D_6_; Me_4_Si) δ (ppm) 41.78 (-CH_2_-), 119.82 (-CH=), 118.92, 121.09, 124.60, 128.21, 129.85, 129.79, 130.36, 130.71, 131.04, 131.42, 139.13 (Ph), 141.36 (-CH=), 15637, 158.59 (Ph), 165.79 (C=O).

Acids and amines, which were weighed in a 1:1.2 ratio, were placed in a flask and connected to the reflux apparatus at 140–160 °C with a 300 W IR light bulb, followed by TLC (hexane: EtOAc, 50:50). Upon completion of the reaction, sufficient AcOEt was added to dissolve the reaction product. A small amount of activated charcoal was added to the reaction mixture and filtered over diatomaceous earth (Hyflo Super Cel^®^ Diatomaceous Earth from Merck KGaA, Darmstadt, Germany) to remove the activated charcoal. Recrystallization from EtOAc produced the corresponding amide (Figure 7). Further recrystallization was performed with an AcOEt or an AcOEt:hexane 90:10 mixture only when necessary.

### 3.2. Body and Liver Weights

**Figure 8:**
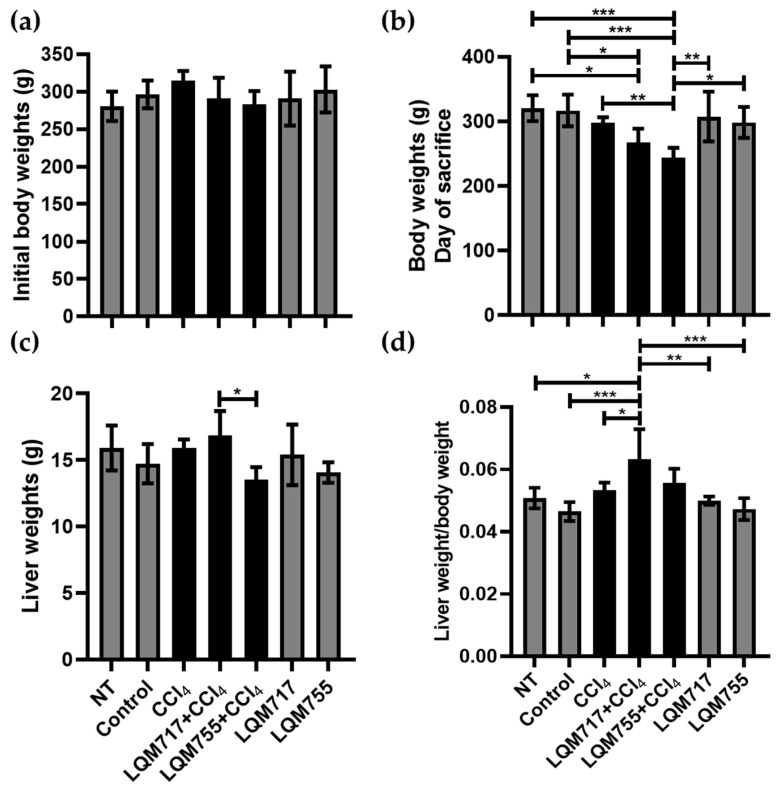
Analysis of body and liver weights. (**a**) Body weights at the beginning of the experiment. (**b**) Body weights on the day of sacrifice. (**c**) Liver Weights after sacrifice. (**d**) Ratio between liver weight and body weight. Results show the average of each group ± SD. Statistically significant differences using a one-way ANOVA with a Tukey–Kramer post hoc test (* *p* < 0.05, ** *p* < 0.01, *** *p* < 0.001).

In the analysis of body weights at the beginning of the treatments, the averages of each experimental group did not differ significantly (Figure 8a). However, on the day of sacrifice (Figure 8b), the healthy groups (NT, Control, LQM717, and LQM755) had similar average weights without significant differences. In contrast, the average weight of the LQM717 + CCl_4_ group was significantly lower than that of the NT (* *p* < 0.05) and Control (* *p* < 0.05) groups. The LQM755 + CCl_4_ group presented a significant reduction in weight compared with the CCl_4_ (** *p* < 0.01), NT (*** *p* < 0.001), Control (*** *p* < 0.001), LQM717 (** *p* < 0.01), and LQM755 (* *p* < 0.05) groups.

However, the average liver weights obtained at sacrifice were significantly lower in the LQM755 + CCl_4_ group in relation to the LQM717 + CCl_4_ group (* *p* < 0.05) (Figure 8c). Finally, the liver/body weight index was greater only in the LQM717 + CCl_4_ group than in the NT (* *p* < 0.05), Control (*** *p* < 0.001), CCl_4_ (* *p* < 0.05), and LQM717 (** *p* < 0.01) and LQM755 (*** *p* < 0.001) groups (Figure 8d).

### 3.3. In Situ Liver Observations

**Figure 9:**
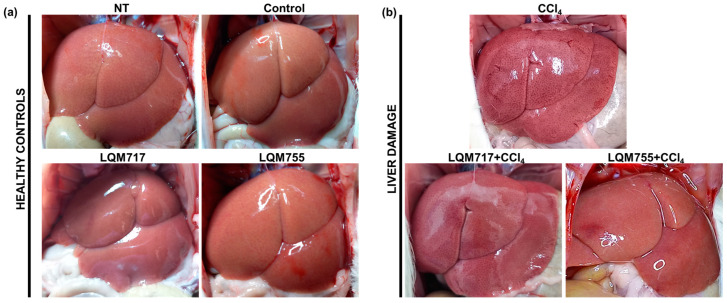
Representative images of livers in situ. (**a**) Healthy controls. (**b**) Group intoxicated with only hepatotoxic CCl_4_ and groups pretreated with LQM717 or LQM755 and intoxicated with CCl_4_.

Macroscopic analysis of the livers of the healthy groups (NT, Control, LQM717, and LQM755) revealed a normal architecture with a uniform red-brown color and a smooth and shiny surface (Figure 9a). In contrast, the CCl_4_ group presented changes in macroscopic morphology, with an opaque brown coloration and numerous necrotic spots on the tissue surface. However, in the groups pretreated with LQM717 or LQM755, the morphological alterations were less pronounced than those in the CCl_4_ group (Figure 9b).

### 3.4. Evaluation of Microscopic Liver Damage via Hematoxylin and Eosin (H&E) Staining

**Figure 10:**
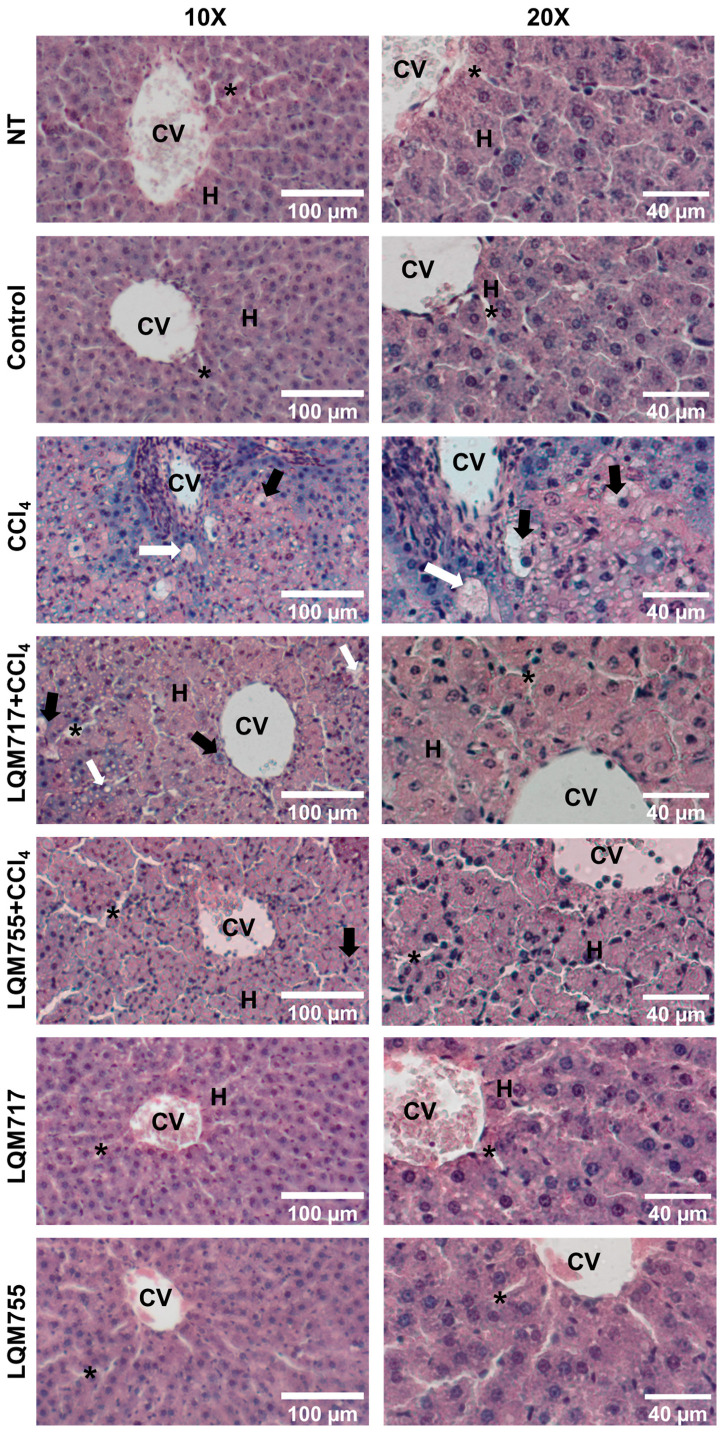
Representative images of histological liver sections stained with H&E. CV = central vein; H = hepatocytes; white arrow = steatosis; black arrow = ballooning necrosis; asterisk = sinusoid.

Histological liver sections stained with H&E (Figure 10) revealed that healthy animals (NT, Control, LQM717, and LQM755 groups) had a normal liver parenchyma without morphological alterations, with sinusoids and cords of hepatocytes radiating from centrilobular veins to the periphery of the lobule. In contrast, the CCl_4_ group presented evident alterations in liver architecture, with a liver parenchyma that presented abundant necrotic lesions and balloon degeneration, characterized by enlarged hepatocytes with granular content due to cytoskeleton collapse, in addition to the presence of steatosis and inflammatory infiltration. However, the LQM717 + CCl_4_ and LQM755 + CCl_4_ groups showed significant prevention, with a notable decrease in necrotic lesions, inflammatory infiltration, and steatosis compared with those of the CCl_4_ group.

### 3.5. Liver Function Tests

Table 3 presents the results of the liver function tests. The serum enzymatic activity of ALT in healthy groups (NT, Control, LQM717, and LQM755) was significantly different (*p* < 0.0001) in relation to the CCl_4_, LQM717 + CCl_4_, and LQM755 + CCl_4_ groups. Furthermore, the LQM717 + CCl_4_ and LQM755 + CCl_4_ groups showed a partial prevention to increased ALT activity, with significant differences compared to the CCl_4_ group (*p* = 0.0045, *p* = 0.0401, respectively).

The enzymatic activity of ALP was partially and significantly lower only in the LQM755 + CCl_4_ group with a statistical significance (*p* < 0.0001) in relation to CCl_4_. On the other hand, all healthy controls showed significant differences (**** *p* < 0.0001) with the CCl_4_ and LQM717 + CCl_4_ groups, while the NT (*p* = 0.0012), LQM717 (*p* = 0.0070), LQM755 (*p* = 0.0030), and Control (*p* = 0.0009) groups were statistically significant with respect to the LQM755 + CCl_4_ group.

GGT serum activity and total bilirubin did not increase in the LQM755 + CCl_4_ group, without significant differences from those of the healthy groups and with significant differences from those of the CCl_4_ group (*p* = 0.0006, *p* = 0.0349, respectively). The LQM717 + CCl_4_ group only showed partial prevention to an increase in GGT activity (*p* = 0.0473) compared to CCl_4_. Direct bilirubin was significantly greater in the CCl_4_ group than in the NT (*p* = 0.0093), Control (*p* = 0.0098), LQM717, and LQM755 (*p* = 0.0085) groups, whereas the LQM717 + CCl_4_ and LQM755 + CCl_4_ groups did not differ significantly from healthy animals and tended to reduce this parameter in relation to the CCl_4_ group.

The serum albumin and liver glycogen levels were significantly lower (*p* < 0.0001) in the CCl_4_ group than in the healthy control groups (NT, Control, LQM717, and LQM755). In the LQM717 + CCl_4_ group, the reduction in serum albumin and liver glycogen contents was partially prevented (*p* = 0.0281, *p* = 0.035, respectively, with respect to CCl_4_). In the LQM755 + CCl_4_ group, the glycogen content reduction was partially prevented compared to the CCl_4_ group (*p* = 0.0400) and healthy controls (*p* < 0.0001), whereas serum albumin reduction was completely prevented. In all tests, healthy animals (NT, Control, LQM717, and LQM755) did not differ significantly.

### 3.6. Detection of Polysaccharides with Periodic Acid-Schiff (PAS) Staining

**Figure 11:**
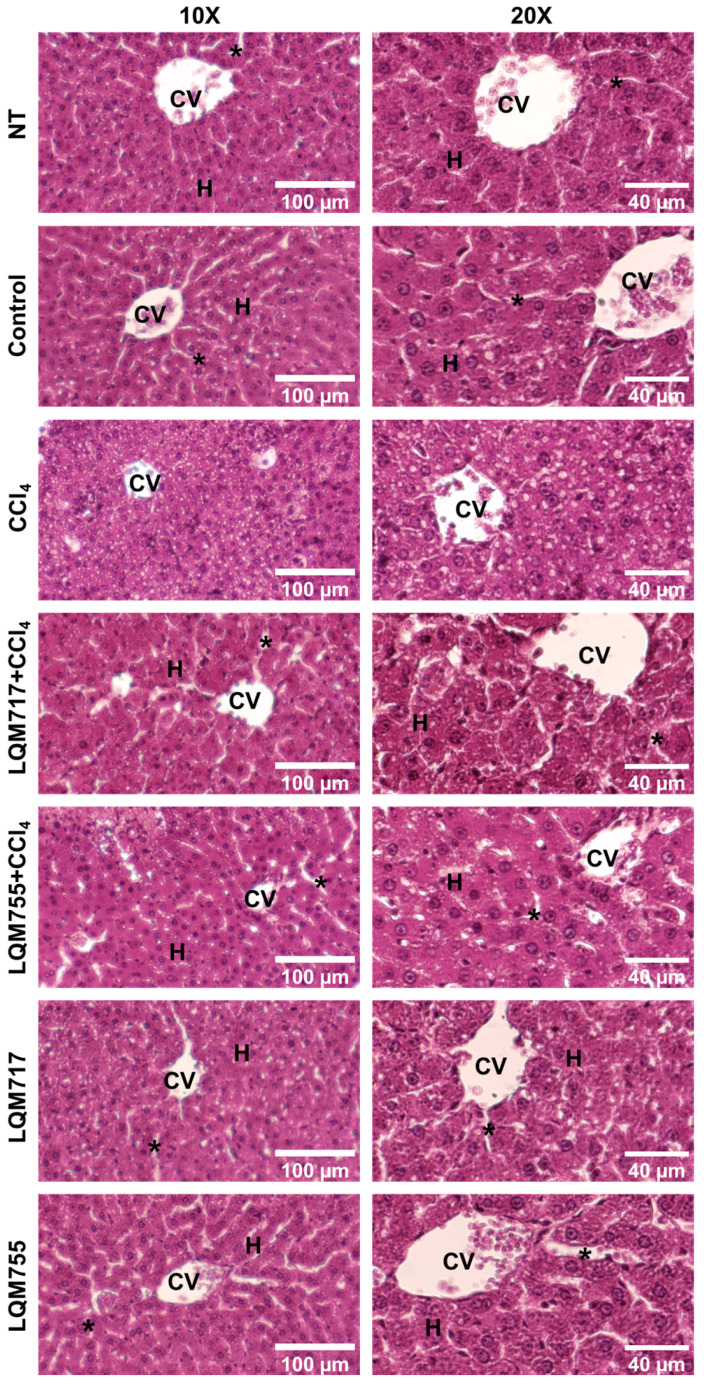
Representative images of histological liver sections stained with PAS. CV = central vein; H = hepatocytes; asterisk = sinusoid.

PAS staining allows the detection of polysaccharides, especially glycogen deposits in liver tissue, which is reflected in a red-purple hue in the tissue [31]. This result is consistent with the biochemical quantification of liver glycogen (Table 3). In the healthy groups (NT, Control, LQM717, and LQM755), an intense and uniform red-purple coloration was observed in the cytoplasm of hepatocytes (Figure 11), suggesting the presence of abundant carbohydrates. In contrast, the tissues of the CCl_4_ group presented a significant decrease in the intensity of the red-purple color, indicating a decrease in carbohydrates in the liver parenchyma compared with those of the healthy groups. Finally, the LQM717 + CCl_4_ and LQM755 + CCl_4_ groups presented preservation of the red-purple hue, suggesting the presence of carbohydrates in the tissue, in contrast to the CCl_4_ group.

### 3.7. Identification of Possible Protein Targets in the Liver

The identification of the possible mechanisms of action of the compounds LQM717 and LQM755 was carried out via five bioinformatic platforms specializing in the prediction of protein targets: Super-PRED (SP), Pharos (P), SEA (S), TargetNet (TN), and Binding DB (BDB). Protein targets expressed in parenchymal and nonparenchymal cells of the liver that perform liver functions or that are prognostic markers in liver cancer were selected. The results allowed the identification of 55 potential protein targets that could interact with the compounds LQM717 and LQM755 in the liver (Table 4, Table 5 and Table 6). Among these genes, 21 were identified as prognostic markers in liver cancer. In total, 32 possible protein targets were identified for compound LQM717 (Table 4 and Table 6), and 36 were identified for compound LQM755 (Table 5 and Table 6), sharing 13 potential protein targets between the two compounds (Table 6).

## 4. Discussion

In the present work, an experimental model of acute liver damage induced by CCl_4_ was established. This model is capable of generating acute liver damage and centrilobular necrosis by administering a single sublethal dose of 4 g/kg orally (p.o.) [44,45]. The results revealed that the ratio between liver weight and body weight in the LQM717 + CCl_4_ group increased significantly, suggesting the presence of hepatomegaly. This condition could be associated with inflammation caused by the hepatotoxic effect of CCl_4_ [46]. In addition, the observed hepatomegaly could also be related to the defense mechanisms used by the body to maintain homeostasis. This process involves an increase in metabolic activity, which is necessary for the repair of damaged tissue [47]. Furthermore, inflammation could be an adaptive response to limit damage and promote liver regeneration.

Importantly, hepatomegaly and inflammation are key indicators of liver response to CCl_4_-induced injury. These findings underscore the relevance of compounds LQM717 and LQM755 in modulating the liver response to damage, suggesting their therapeutic potential in the prevention and treatment of toxin-induced liver diseases.

Macroscopic (Figure 9) and microscopic (Figure 10 and Figure 11) observations of the livers revealed that the compounds LQM717 and LQM755 reduce acute liver damage induced by a sublethal dose of CCl_4_. These observations are consistent with the results of the liver function tests (Table 3). The NT and Control groups, as well as the groups treated with LQM717 and LQM755, did not differ significantly, suggesting that at the doses used in this study, these compounds do not have hepatotoxic effects.

In contrast, the CCl_4_-treated group presented significant increases in ALT, ALP, and GGT enzymatic activity, as well as total and direct bilirubin levels, and a decrease in serum albumin and liver glycogen content. This is because CCl_4_ generates acute toxicity through its metabolism mediated by CYP450, producing free radicals such as the trichloromethyl radical (CCl_3_˙) and the trichloromethylperoxy radical (CCl_3_OO ˙), which react with proteins, nucleic acids, and lipids [46].

Pretreatment with LQM717 or LQM755 significantly prevented CCl_4_-induced liver damage. However, differences were observed between the two compounds derived from cinnamic acid (Table 3). LQM755 showed greater hepatoprotective activity than LQM717 since it partially but significantly prevented increases in ALT and ALP, whereas LQM717 only showed partial ALT activity. Further, LQM755 demonstrated a higher decrease in cholestatic biomarkers than LQM717, completely preventing an increase in GGT, whereas LQM717 only partially did so. Total bilirubin increase was also prevented with LQM755 pretreatment, but not with LQM717.

Cinnamic acid (20 mg/kg) has been shown to attenuate cisplatin-induced hepatotoxicity, resulting in partial prevention of increased total serum bilirubin [48]. Furthermore, at a dose of 50 mg/kg, the serum enzymatic activity of ALT, but not that of ALP, decreased in rats treated with gentamicin [23].

Considering that serum albumin is synthesized exclusively by hepatocytes and that its levels tend to decrease in chronic liver diseases and in cases of severe acute liver damage [49], the results obtained indicate that LQM755 could be a promising drug for the treatment of hepatic cholestasis; therefore, further studies will be required in cholestasis models, such as the bile duct ligation model in rats. This compound completely prevented the reduction in the serum albumin concentration, while LQM717 only partially did so. These findings suggest that LQM755 helps maintain liver function despite CCl_4_-induced damage.

Evaluation of liver biosynthetic capacity includes the quantification of liver glycogen and histological evaluation via PAS staining. Our results indicate that both LQM717 and LQM755 significantly prevent CCl_4_-induced carbohydrate depletion. These findings are related to those of a previous study with cinnamic acid (50 mg/kg), which only modestly prevented liver glycogen depletion in experimental animals challenged with CCl_4_ [25]. In the present work, the results for serum albumin and liver glycogen suggest that LQM717 and LQM755 help to preserve the biosynthetic capacity of the liver on acute liver damage, a condition not previously reported. The hepatoprotective effects described above may be related to previous studies evaluating the biological activity of these compounds. LQM717 prevented diethylnitrosamine-induced necrosis in a resistant hepatocyte modified model in rats [19]. LQM755 has shown antineoplastic activity by inhibiting breast cancer growth in the MCF-7 cell line and lung cancer [50].

Since the mechanism of action of LQM717 and LQM755 is still unknown, bioinformatic platforms were used to predict possible target proteins in the liver (Table 4, Table 5 and Table 6). Among the results obtained for LQM717 (Table 4), the following stand out:

(a) Carboxylesterase 1 (CES1, P23141) is involved in the detoxification of xenobiotics, and its inhibition is beneficial for the treatment of metabolic disorders, such as obesity and fatty liver disease [51];
(b) CYP450 3A4 (P08684) is considered an important CYP450 whose function is to detoxify bile acids, and the stimulation of its activity could be useful for the treatment of cholestatis [52];
(c) The DNA excision repair protein ERCC-1 (P07992) may influence survival time after chemotherapy [53].

In the case of LQM755 (Table 5), the following targets stand out:

(a) MMP-1 (P03956);
(b) MMP-9 (P14780);
(c) MMP-12 (P39900).

Chronic liver damage promotes fibrosis progression, a pathological condition that involves mainly the activation of liver stellate cells, which leads to excessive accumulation of the extracellular matrix (ECM) [54]. Matrix metalloproteinases (MMPs) play complex roles in modulating ECM degradation. Increasing MMP-1 reduces fibrosis progression by degrading the fibrillar ECM; MMP-9 is a matrix-degrading enzyme expressed in the early stages of liver damage through nuclear factor kappa-B (NF-κB) and mitogen-activated protein kinase, and MMP-12 facilitates the degradation of elastin [55].

In particular, NF-κB (Q04206) is another potential target protein of LQM755 (Table 5). This nuclear transcription factor is closely related to liver damage, and its inhibition delays the inflammatory response and oxidative stress and ultimately reduces hepatocellular death [56]. In addition, 13 possible target proteins were found that coincided with both compounds of interest (Table 6), including the Nuclear factor-erythroid 2 related factor 2 (Nrf2, Q16236). Dysregulation of Nrf2 signaling is related to oxidative stress and inflammation in diseases such as neurological diseases, diabetes mellitus, liver diseases, and cancer [57,58,59,60]. Therefore, there is a complex and bidirectional relationship between NF-κB and Nrf2, because these factors regulate opposite cellular pathways. On the one hand, NF-κB is a master regulator of inflammation, immune responses, and cell survival [61]. On the other hand, Nrf2 is a master transcriptional regulator of antioxidant and detoxification responses [62]. This could partly explain the superior effect of LQM755 compared to LQM717, as shown in Table 5 and Table 6; NF-κB and Nrf2 are potential protein targets for LQM755, perhaps modulating the activation of the Nrf2 antioxidant pathway and, in turn, inhibiting the NF-κB pro-inflammatory pathway. It is important to highlight that the potential therapeutic targets were identified through bioinformatic analysis and remain to be experimentally validated in future research.

Notably, the present work showed similarities between the hepatoprotective action of both compounds; LQM717 and LQM755 partially prevent increases in ALT enzymatic activity. However, LQM755 stands out by completely preventing an increase in GGT and total bilirubin, while LQM717 only partially prevents an increase in GGT. Likewise, it is highlighted that LQM755 maintains serum albumin production and significantly reduces glycogen depletion, while LQM717 only partially maintains both parameters.

## 5. Conclusions

The results of our study indicate that synthetic cinnamic acid analogs, specifically LQM717 and LQM755, have the ability to significantly prevent CCl_4_-induced acute liver damage. These compounds demonstrated remarkable hepatoprotection, in addition to maintaining the biosynthetic capacity of the liver. In particular, the compound LQM755 showed greater efficacy in the prevention of acute hepatocellular damage induced by CCl_4_. Additionally, bioinformatic analysis revealed several possible protein targets, which opens a wide range of opportunities to explore and elucidate the mechanisms of action and the signaling pathways responsible for the observed hepatoprotective effects. However, these compounds must be evaluated in subsequent studies for their potential anti-inflammatory, antifibrotic, and antisteatotic effects to determine their hepatoprotective potential and action mechanism. Therefore, these early findings are expected to promote further research on the hepatoprotective activity of both compounds using others acute and chronic models of liver damage. Further studies could ultimately contribute to the development of effective drug treatments for liver diseases.

## Figures and Tables

**Table 1:** Physicochemical characteristics of LQM717 and LQM755.

Compound	LQM717	LQM755
Chemical structure	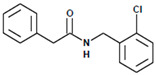	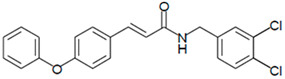
Chemical name	N-(2-chlorobenzyl)-2-phenylacetamide	N-[(3,4-dichlorophenyl)methyl]-3-(4-phenoxyphenyl)-2-propenamide
Molecular weight	259.73 g/mol	398.29 g/mol
Physical characteristics	White solid	White crystalline solid

**Table 2:** Intraperitoneal dosing of LQM717 and LQM755.

Compound	Dose	Vehicle
**LQM717**	20 mg/kg	100 μL de DMSO
**LQM755**	LQM717 equimolar dose	100 μL de DMSO

**Table 3:** Biochemical tests on serum and liver samples.

	Serum Samples						Liver Samples
	ALT	ALP	GGT	Total Bilirubin	Direct Bilirubin	Albumin	Glycogen
NT	36.56 ± 16.61 ^abc****^	35.24 ± 6.072 ^ab****c**^	14.13 ± 9.972 ^a****b**^	0.97 ± 0.326 ^a**b*^	0.47 ± 0.239 ^a**^	578.39 ± 24.11 ^a****b**^	2.08 ± 0.08 ^abc****^
Control	39.92 ± 12.01 ^abc****^	33.60 ± 3.691 ^ab****c***^	7.88 ± 5.765 ^a****b**^	0.76 ± 0.679 ^a**b*^	0.56 ± 0.498 ^a**^	562.69 ± 20.3 ^a****b*^	1.91 ± 0.15 ^abc****^
CCl_4_	184.07 ± 14.83 ^b**c*^	253.22 ± 11.94 ^c****^	210.34 ± 65.01 ^b*c***^	25.86 ± 9.24 ^c*^	15.3 ± 9.372	471.73 ± 19.0 ^b*c**^	0.44 ± 0.02 ^bc*^
LQM717 + CCl_4_	151.46 ± 14.65 ^a**^	220.92 ± 42.72	128.59 ± 58.4 ^a*^	25.47 ± 15.37	11.39 ± 4.644	509.08 ± 14.05 ^a*^	0.62 ± 0.11 ^a*^
LQM755 + CCl_4_	161.21 ± 10.87 ^a*^	109.12 ± 35.86 ^a****b****^	90.49 ± 32.76 ^a***^	8.16 ± 6.24 ^a*^	6.27 ± 3.306	531.30 ± 18.07 ^a**^	0.64 ± 0.057 ^a*^
LQM717	47.69 ± 8.044 ^abc****^	46.31 ± 3.743 ^ab****c**^	12.85 ± 10.68 ^a****b**^	0.54 ± 0.49 ^a**b**^	0.32 ± 0.552 ^a**^	575.97 ± 29.61 ^a****b**^	2.02 ± 0.24 ^abc****^
LQM755	51.48 ± 4.022 ^abc****^	40.93 ± 3.737 ^ab****c**^	15.14 ± 10.48 ^a****b**^	1.63 ± 0.65 ^a**b*^	0.31 ± 0.36 ^a**^	555.45 ± 10.43 ^a****b*^	1.98 ± 0.08 ^abc****^

Results show the average of each group ± SD. One-way ANOVA analysis: ALT (F 130.2, **** *p* < 0.0001); ALP (81.91, **** *p* < 0.0001); GGT (F 20.13, **** *p* < 0.0001); Total bilirubin (F 8.215, **** *p* < 0.0001); Direct bilirubin (F 6.310, *** *p* < 0.0007); Albumin (F 16.83, **** *p* < 0.0001), and Glycogen (F 183.4, **** *p* < 0.0001) and Tukey–Kramer post hoc test with statistically significant differences (* *p* < 0.05, ** *p* < 0.01, *** *p* < 0.001, **** *p* < 0.0001). Letters indicate which groups are significantly different: a: with respect to CCl_4_; b: with respect to LQM717 + CCl_4_; c: with respect to LQM755 + CCl_4_.

**Table 4:** Bioinformatic analysis of putative protein targets to LQM717 in liver.

Target Protein Name	Protein ID	Target Location and/or Function Human Protein Atlas	Database
Alanyl aminopeptidase	P15144	Enriched cell type: hepatocytes/Protease angiogenesis	TN
Bifunctional epoxide hydrolase 2	P34913	Enriched cell type: hepatocytes/Lipid and xenobiotic metabolism	TN
17-beta-hydroxysteroid dehydrogenase type 2	P37059	Enriched cell type: hepatocytes and cholangiocytes/Lipid metabolism	BDB
Carbonic anhydrase 2	P00918	Enriched cell type: hepatocytes/Lyase	S
Carbonic anhydrase 5A, mitochondrial	P35218	Enriched cell type: hepatocytes/Ureagenesis	S, SP
Carbonic anhydrase 9 *	Q16790	Enriched cell type: cholangiocytes/Lyase	S
Carboxylesterase 1	P23141	Enriched cell type: hepatocytes/Lipid metabolism	TN
Carboxylesterase 2	O00748	Enriched cell type: hepatocytes/Lipid metabolism	TN
CYP450 2C9 *	P11712	Enriched cell type: hepatocytes/Lipid metabolism	S
CYP450 3A4	P08684	Enriched cell type: hepatocytes/Lipid metabolism	S
Coagulation factor II, thrombin	P00734	Enriched cell type: hepatocytes/Hemostasis	TN
DNA excision repair protein ERCC-1 *	P07992	Liver/DNA repair	S
Folate hydrolase 1 *	Q04609	Enriched cell type: hepatocytes/Multifunctional enzyme	TN
Histone deacetylase 1 *	Q13547	Liver/Transcription regulation	P, S
Mitogen-activated protein kinase 8 *	P45983	Liver/Proliferation and programmed cell death	P, S, TN
Perilipin-1	O60240	Enriched cell type: Hepatocytes/Lipid metabolism	TN
Perilipin-5 *	Q00G26	Enriched cell type: Hepatocytes/Lipid metabolism	TN
Proto-oncogene tyrosine-protein kinase Src *	P12931	Liver/Cell cycle	BDB
Solute carrier family 2, facilitated glucose transporter member 1 *	P11166	Liver/Glucose transporter	SP

TN: TargetNet; BDB: Binding DB; S: SEA; SP: Super-PRED; P: Pharos. (*): prognostic marker in liver cancer.

**Table 5:** Bioinformatic analysis of putative protein targets to LQM755 in liver.

Target Protein Name	Protein ID	Target Location and/or Function Human Protein Atlas	Database
Amine oxidase [flavin-containing] B	P27338	Enriched cell type: hepatocytes/Oxidative amine deamination	SP, TN
Broad substrate specificity ATP-binding cassette transporter ABCG2	Q9UNQ0	Enriched cell type: hepatocytes/Lipid transport	TN
C-C chemokine receptor type 2	P41597	Enriched cell type: Kupffer cells/Inflammatory response	TN
C5a anaphylatoxin chemotactic receptor 1	P21730	Enriched cell type: Kupffer cells/Chemotaxis	SP
Dihydroorotate dehydrogenase (quinone), mitocondrial	Q02127	Enriched cell type: hepatocytes/Pyrimidine biosynthesis	SP
Glycogen phosphorylase, liver form	P06737	Enriched cell type: hepatocytes/Carbohydrate metabolism	P, S, TN
Histone deacetylase 2 *	Q92769	Liver/Transcription regulation	TN
Histone deacetylase 5 *	Q9UQL6	Liver/Transcription regulation	SP
Histone deacetylase 7	Q8WUI4	Liver/Transcription regulation	SP
Matrix metalloproteinase-1 (MMP-1) *	P03956	Enriched cell type: Cholangiocytes/Collagen degradation	P, S, TN
Matrix metalloproteinase-2 (MMP-2)	P08253	Enriched cell type: hepatic stellate cells/Angiogenesis and collagen degradation	BDB, P, S, TN
Matrix metalloproteinase-9 (MMP-9) *	P14780	Liver/Collagen degradation	BDB, P, S, TN
Matrix metalloproteinase-12 (MMP-12) *	P39900	Liver/Metalloprotease	TN
Nuclear factor NF-kappa-B p65 subunit *	Q04206	Liver/NF-kB Subunit	TN
Nuclear receptor subfamily 1 group H member 4 (Bile acid receptor)	Q96RI1	Enriched cell type: hepatocytes and cholangiocytes/Bile acid transport	TN
Peroxisome proliferator-activated receptor Alpha	Q07869	Enriched cell type: hepatocytes/Transcription regulation	TN
Peroxisome proliferator-activated receptor gamma *	P37231	Liver/Transcription regulation	TN
Polyunsaturated fatty acid 5-lipoxygenase	P09917	Enriched cell type: Kupffer cells/Leukotriene biosynthesis	TN
Proteasome subunit beta type-2 *	P49721	Liver/Protein degradation	SP
Transient receptor potential cation channel subfamily V member 4	Q9HBA0	Enriched cell type: Kupffer cells and cholangiocytes/Ion transport	BDB
Transthyretin	P02766	Enriched cell type: hepatocytes/Transport thyroid hormones	SP
Tyrosyl-DNA phosphodiesterase 1 *	Q9NUW8	Liver/DNA repair	SP
Vascular cell adhesion protein 1	P19320	Enriched cell type: Kupffer cells/Cell adhesion	TN

SP: Super-PRED; TN: TargetNet; S: SEA; P: Pharos; BDB: Binding DB. (*): prognostic marker in liver cancer.

**Table 6:** Bioinformatic analysis of putative protein targets that could interact with both LQM717 and LQM755 in liver.

Target Protein Name	Protein ID	Target Location and/or Function Human Protein Atlas	Database
Apurinic/Apyrimidinic Endodeoxyribonuclease 1 *	P27695	Liver/DNA repair and transcription regulation	SP
CYP450 2C19	P33261	Enriched cell type: hepatocytes/Lipid metabolism	TN, S
Histone deacetylase 6	Q9UBN7	Enriched cell type: hepatocytes/Transcription regulation	P, S, TN
M-phase inducer phosphatase 2 *	P30305	Liver/Cell cycle	TN, SP
Nuclear factor erythroid 2-related factor 2	Q16236	Liver/Antioxidant response	SP
Nuclear factor NF-kappa-B p105 subunit	P19838	Liver/NF-kB subunit	SP
Nuclear receptor subfamily 1 group I member 2	O75469	Enriched cell type: hepatocytes/Xenobiotic and drug metabolism	SP
Prostaglandin E2 receptor EP2 subtype	P43116	Enriched cell type: Kupffer cells/G protein-coupled receptor	TN
Prostaglandin-endoperoxide synthase 1	P23219	Enriched cell type: hepatic stellate cells/Lipid and prostaglandin metabolism	SP
Sigma nonopioid intracellular receptor 1	Q99720	Enriched cell type: hepatocytes/Lipid transport	S, TN
Tachykinin receptor 2	P21452	Enriched cell type: Kupffer cells/G protein-coupled receptor	SP
Transcription intermediary factor 1-alpha *	O15164	Liver/Regulation of cell proliferation	SP
Transient receptor potential cation channel subfamily V member 1	Q8NER1	Enriched cell type: hepatocytes/Ion transport	BDB

SP: Super-PRED; TN: TargetNet; S: SEA; P: Pharos; BDB: Binding DB. (*): prognostic marker in liver cancer.

## Data Availability

The data presented in this study are available on request from the corresponding author J.R.M.-P.
